# Characterization of injury in isolated rat proximal tubules during cold incubation and rewarming

**DOI:** 10.1371/journal.pone.0180553

**Published:** 2017-07-03

**Authors:** Anja Bienholz, Björn Walter, Gesine Pless-Petig, Hana Guberina, Andreas Kribben, Oliver Witzke, Ursula Rauen

**Affiliations:** 1 Department of Nephrology, University Hospital Essen, University Duisburg-Essen, Essen, Germany; 2 Institute of Physiological Chemistry, University Hospital Essen, University Duisburg-Essen, Essen, Germany; 3 Department of Infectious Diseases, University Hospital Essen, University Duisburg-Essen, Essen, Germany; Virginia Commonwealth University, UNITED STATES

## Abstract

Organ shortage leads to an increased utilization of marginal organs which are particularly sensitive to storage-associated damage. Cold incubation and rewarming-induced injury is iron-dependent in many cell types. In addition, a chloride-dependent component of injury has been described. This work examines the injury induced by cold incubation and rewarming in isolated rat renal proximal tubules. The tissue storage solution TiProtec^®^ and a chloride-poor modification, each with and without iron chelators, were used for cold incubation. Incubation was performed 4°C for up to 168 h, followed by rewarming in an extracellular buffer (3 h at 37°C). After 48, 120 and 168 h of cold incubation LDH release was lower in solutions containing iron chelators. After rewarming, injury increased especially after cold incubation in chelator-free solutions. Without addition of iron chelators LDH release showed a tendency to be higher in chloride-poor solutions. Following rewarming after 48 h of cold incubation lipid peroxidation was significantly decreased and metabolic activity was tendentially better in tubules incubated with iron chelators. Morphological alterations included mitochondrial swelling and fragmentation being partially reversible during rewarming. ATP content was better preserved in chloride-rich solutions. During rewarming, there was a further decline of ATP content in the so far best conditions and minor alterations under the other conditions, while oxygen consumption was not significantly different compared to non-stored control tubules. Results show an iron-dependent component of preservation injury during cold incubation and rewarming in rat proximal renal tubules and reveal a benefit of chloride for the maintenance of tubular energy state during cold incubation.

## Introduction

Solid organ transplantation in general, and kidney transplantation in particular are torn between the conflicting areas of organ supply and demand. The currently existing shortage of kidney donations and the steadily rising number of potential recipients dying while being on the waiting list encourages transplantation of marginal organs by accepting older and expanded criteria donors [1, 2]. Marginal organs are particularly sensitive to storage-associated damage with an increasing risk for delayed or poor graft function affecting long-term organ survival with effects on both patients`morbidity and mortality [3, 4]. Therefore, improvements of currently used storage and transport conditions are urgently needed to maintain and enhance organ quality and thereby patient outcome.

Discontinuity of blood flow during kidney explantation is followed by a lack of oxygen supply which massively impairs mitochondrial energy production. Renal proximal tubules are especially sensitive to hypoxia as they require energy for multiple transport processes and are highly dependent on aerobic energy metabolism for ATP synthesis due to their enzymatic equipment [5]. Mitochondrial energy production cannot be entirely restored even after full reoxygenation [6].

During transportation organs are usually stored in special preservation solutions on ice. Hypothermia decreases hypoxia-induced injury, but triggers cell damage by itself [7–9]. Furthermore, as organ quality is currently rather poor there are approaches to replace conventional simple (ischemic) cold storage of kidneys by machine perfusion [10, 11]. During machine perfusion kidneys are perfused continuously with a likewise hypothermic, but oxygen-containing preservation solution leading to decline of ischemic damage leaving hypothermia as the primary (and in the presence of oxygen even aggravated, see below) trigger of injury.

According to the “classic” hypothesis on cold-induced injury hypothermia leads to inhibition of the Na^+^/K^+^-ATPase resulting in intracellular sodium accumulation with accompanying chloride influx [12, 13]. Changes in intracellular osmolarity are thought to cause subsequent water influx leading to cellular edema and cell damage. Therefore, many clinically used preservation solutions contain little or no chloride. In the University of Wisconsin solution (UW) [12] predominantly used in the USA, chloride is completely substituted by the impermeable anion lactobionate, while gluconate is the anion of choice in the KPS1 solution used for machine perfusion [14]. The histidine-tryptophan-ketoglutarate solution (HTK) [15], which is often used for kidney storage in the Eurotransplant area, contains 50 mM chloride being far below the physiological extracellular chloride concentration. However, in contradiction to the “classic” hypothesis, hepatocytes and endothelial cells do not show sodium accumulation or cellular swelling when stored in sodium-containing solutions in the cold [9, 16, 17]. In line with these results sodium-free media do not inhibit cold-induced injury of hepatocytes and the kidney cell line LLC-PK_1_ [9].

Meanwhile, an increase in cellular chelatable iron ions causing formation of highly reactive oxygen species (ROS) has been identified as the main component of cold-induced cellular damage in different cell types. The cold-induced increase in cellular/cytosolic chelatable iron ions has been shown to trigger lipid peroxidation and mitochondrial permeability transition [7, 18, 19]. This iron-dependent component of cold-induced injury has also been described for renal tubular cells and tubules [8, 19–21] and can effectively be blocked by addition of iron chelators to preservation solutions [8, 21].

However, in cells protected from the iron-dependent component of damage further iron-*in*dependent components come to the fore from part of which cells are also not protected by conventional preservation solutions. In particular, a chloride-dependent component of injury has been described in rat hepatocytes [16, 22], while chloride-poor solutions accentuated damage in endothelial cells [23, 24]. The role of chloride in cold-induced injury of renal tubules has not yet been addressed.

The current study therefore characterizes cold-induced injury to renal tubules in a model of isolated rat proximal tubules with a focus on the role of chloride (or its absence) in this injury.

## Materials and methods

Chemicals and Materials. Cold incubation solutions (composition see Table 1) are based on the tissue storage solution TiProtec^®^ [23], a derivative of the new preservation solution Custodiol-N. This tissue preservation solution was used here, as it can easily be modified with regard to the chloride content, yielding a high chloride and a low chloride cold storage solution [16, 23]. The term “iron chelators” refers to 0.5 mM deferoxamine plus 20 μM LK 614. This combination of the strong, but large and hydrophilic and thus relatively poorly membrane-permeable chelator deferoxamine (that, however, does enter cells [25]) and the small lipophilic hydroxamic acid derivative LK 614 [23, 26] has been shown previously to provide optimal protection and to affect intracellular iron-dependent processes [16, 27]. The extra-cellular buffer was used in previous experiments with isolated renal proximal tubules [28, 29].

**Table 1:** Composition of cold incubation solutions and of extra-cellular buffer used during rewarming.

	Solution 1	Solution 2	Solution 1 + chelators	Solution 2 + chelators	Extra-cellular buffer
**Cl**^**-**^	103	8	103	8	113
**α-Ketoglutarate**	2	2	2	2	-
**Aspartate**	5	5	5	5	-
**Lactobionate**	-	95	-	95	-
**H**_**2**_**PO**_**4**_^**-**^	1	1	1	1	2
**SO**_**4**_^**2-**^	-	-	-	-	1
**HCO**_**3**_^**-**^	-	-	-	-	20
**Na**^**+**^	16	16	16	16	142
**K**^**+**^	93	93	93	93	5
**Mg**^**2+**^	8	8	8	8	1
**Ca**^**2+**^	0.05	0.05	0.05	0.05	1
**HEPES**	-	-	-	-	10
***N*-Acetylhistidine**	30	30	30	30	-
**Glycine**	10	10	10	10	2
**Alanine**	5	5	5	5	1
**Tryptophan**	2	2	2	2	-
**Sucrose**	20	20	20	20	-
**Glucose**	10	10	10	10	5
**Lactate**	-	-	-	-	4
**Butyrate**	-	-	-	-	10
**Deferoxamine**	-	-	0.5	0.5	-
**LK 614**	-	-	0.02	0.02	-
**pH**	7.0	7.0	7.0	7.0	7.2
**Osmolarity (mosm/l)**	305	305	306	306	317

Concentrations are given in mmol/l, calculated osmolarity in mosm/l. pH was adjusted at room temperature.

Animals. Male Sprague Dawley rats (250–320 g) were obtained from Charles River (Sulzfeld, Germany). Animals were kept for at least one week prior to the experiments in the central animal unit of the University Hospital Essen under standardized conditions of temperature (22 ± 1°C), humidity (55 ± 5%) and 12 h/12 h light/dark cycles with free access to food (sniff-Spezialdiäten, Soest, Germany) and water; animals were not fasted prior to the experimental procedure. All animals received humane care according to the standards of the Federation of European Laboratory Animal Science Association (FELASA). The experimental protocol has been approved based on the German animal protection act by the state office for nature, environment and consumer protection (LANUV Recklinghausen, AZ 84–02.04.2014.A001).

Isolation of renal proximal tubules. Renal proximal tubules were freshly isolated as described previously with slight modification [29]. Rats were anaesthetized with xylazine and ketamine (Ceva, Düsseldorf, Germany) (6 and 120 mg/kg bw i.p.). Kidneys were flushed through an aortic catheter with 40 ml of oxygenated buffered salt solution (solution A) containing (in mM) NaCl 112; NaHCO_3_ 20; KCl 5; CaCl_2_ 1.6; NaH_2_PO_4_ 2; MgSO_4_ 1.2; glucose 5; HEPES 10; mannitol 10; glutamine 1; sodium butyrate 1; sodium lactate 1; pH 7.05, 4°C with the addition of 2000 I.E. heparin (Ratiopharm GmbH, Ulm, Germany). Subsequently, perfusion was continued with 30 ml of oxygenated solution A containing 5 mg of collagenase (type A, specific activity 0.228 U/mg; Lot 70128622, Roche Diagnostics GmbH, Mannheim, Germany) and 37.5 mg of hyaluronidase (300 U/mg; Lot 131169777, Carl Roth GmbH, Karlsruhe, Germany). After perfusion, the kidneys were decapsulated, removed and transferred to ice-cold solution A. The renal cortices were dissected and minced on a cold petri dish, then incubated for 30 min in 30 ml of oxygenated solution A containing 10 mg collagenase and 22.5 mg of hyaluronidase for digestion. Separation of the tubules was checked regularly by microscopy. Separated tubules were washed and placed in 30 ml of ice-cold solution A containing 1 g of bovine serum albumin (Serva, Heidelberg, Germany) for 20 min. After filtering the tissue and two washes to remove albumin, the tubules were separated and enriched using centrifugation on self-forming Percoll gradients as previously described [28]. Proximal tubules were resuspended in 50 ml and washed three times to remove the Percoll.

Tubules were equally distributed to six flasks of which 4–5 contained the indicated pre-cooled solutions and were directly used for cold incubation experimental procedures. The other 1–2 flasks always contained 5 ml of pre-cooled extra-cellular buffer. As described previously, these flasks were kept at room temperature for 10 min followed by 10 min at 37°C.[29] After warming a sample for measurement of baseline ATP was taken or baseline imaging or baseline measurements of resazurin reduction or of respiration were performed (see below).

Cold incubation. Incubation was performed in flasks filled with 5 ml of indicated pre-cooled solutions at 4°C under gentle motion for the indicated span of time.

Rewarming. For gentle rewarming tubule suspensions in the respective cold solutions were kept at room temperature for 10 min followed by 10 min at 37°C (transition period). After that tubules were spun down, resuspended in 5 ml pre-warmed extra-cellular buffer and incubated under gentle motion at 37°C (5% CO_2_, humidified atmosphere) for three hours.

ATP content. Suspension samples of 0.5 ml were added to 50 μl of 10 M perchloric acid, vigorously mixed and immediately frozen in liquid nitrogen and stored at -80°C. ATP content was measured using a luciferase-driven bioluminescence assay (ATP Bioluminescence Assay Kit CLS II, Roche, Mannheim, Germany). After thawing, samples were diluted 1:10,000 in buffer containing 100 mM TRIS and 4 mM EDTA (pH 7.75) and mixed immediately with luciferase reagent. Light emission was detected at 550 nm using a luminometer (Berthold Detection Systems, Pforzheim, Germany). The protein content was determined according to Lowry [30]. ATP content normalized for protein content is expressed as percentage of the respective non-stored control tubules.

LDH release. Release of the cytosolic enzyme lactate dehydrogenase (LDH), which can only be detected extracellularly after destruction of the cellular plasma membrane, was used as a robust and quantitative marker of cell death. LDH was measured photometrically using a standard assay. At indicated time points LDH activity was measured in supernatants of the tubule suspension. In addition, residual intracellular LDH was determined after lysis of the tubules with Triton X-100 (1% in Hanks`balanced salt solution, 30 min at 37°C). Results display released LDH activity as a percentage of total (i.e. of residual intracellular plus extracellular) LDH activity.

Thiobarbituric acid-reactive substances. Thiobarbituric acid-reactive substances (TBARS) were determined in supernatant at the end of cold incubation and in the extracellular buffer at the end of rewarming using the assay described in reference [31] with minor modifications (allowing a sample volume of 1 ml). Blanks of all solutions were included. The amounts of TBARS formed were determined as malondialdehyde equivalents using 1,1,3,3-tetramethoxy-propane as a standard and are expressed in relation to the average protein content of two parallel tubule suspension aliquots (of the same tubule isolation) taken at time zero.

Immunoblotting. For Western Blotting, cell extracts were prepared in RIPA buffer (50 mM Tris-HCl pH 8.0, 150 mM sodium chloride, 0.5% (w/v) sodium deoxycholate, 0.1% (w/v) SDS) supplemented with protease/phosphatase inhibitor cocktail (Cell Signaling, #5872) by mechanical homogenization, incubation on ice (30 min) and sonication (5 min). Insoluble materials were removed (27.000 g, 15 min, 4°C). Protein concentration was determined using Pierce BCA Protein Assay Kit (Thermo Scientific). Equal amounts of proteins were separated by denaturing 15% SDS-PAGE and either visualized by coomassie staining (loading control) or transferred onto nitrocellulose membrane (Amersham Protran 0.2 μm, GE Healthcare). Full-length and cleaved caspase 3 were detected using a polyclonal rabbit antibody against caspase 3 (Cell Signaling, #9662, used according to the manufacturer´s suggestions) and a horseradish peroxidase-conjugated goat anti-rabbit IgG secondary antibody (Sc-2004, Santa Cruz). Signals were visualized using ECL reaction (SuperSignal West Femto, Thermo Scientific) and quantified using ImageJ program (https://imagej.nih.gov/ij/). Cleaved caspase 3 of cold-stored tubules is given as percentage of the respective signal of non-stored control tubules.

Resazurin reduction. Conversion of resazurin to resorufin, a general indicator for reductive cell metabolism, was determined as described previously [26]. Conversion rate was calculated as percentage of non-stored control tubules.

Cellular respiration. Respiration was measured at baseline and after 48 h of cold incubation in solutions 1+chelators and 2+chelators followed by 2 h or 3 h of rewarming in extracellular buffer. Respiration was measured in 2.5 ml extra-cellular buffer at 37°C under continuous stirring using an O2K-oxygraph (Oroboros Instruments, Innsbruck, Austria). Respiration is given for an aliquot of tubules corresponding to about 2 mg of protein.

Imaging. Samples of the tubule suspensions were stained with 300 nM Mito Tracker Red CMXRos (Life Technologies GmbH, Darmstadt, Germany) at the start of the experiments (37°C, 20 min for baseline or throughout cold incubation at 4°C), and fixed with 3.7% paraformaldehyde in M 199 cell culture medium with 20% fetal calf serum immediately (37°C, 15 min, baseline), and at the end of 48 h cold incubation (4°C, 10 min) or after 3 h rewarming (37°C, 15 min). Confocal images were obtained using a Zeiss ELYRA PS 1 microscope with LSM 710 confocal laser scanning unit (Zeiss, Oberkochen, Germany).

Statistics. Experiments were performed repeatedly as indicated in figure legends. Data are expressed as mean values ± standard deviation. Comparison among multiple groups was performed using analysis of variances (ANOVA) followed by Bonferroni or Dunns post-hoc analysis. A p-value < 0.05 was considered statistically significant.

## Results

**Fig 1:**
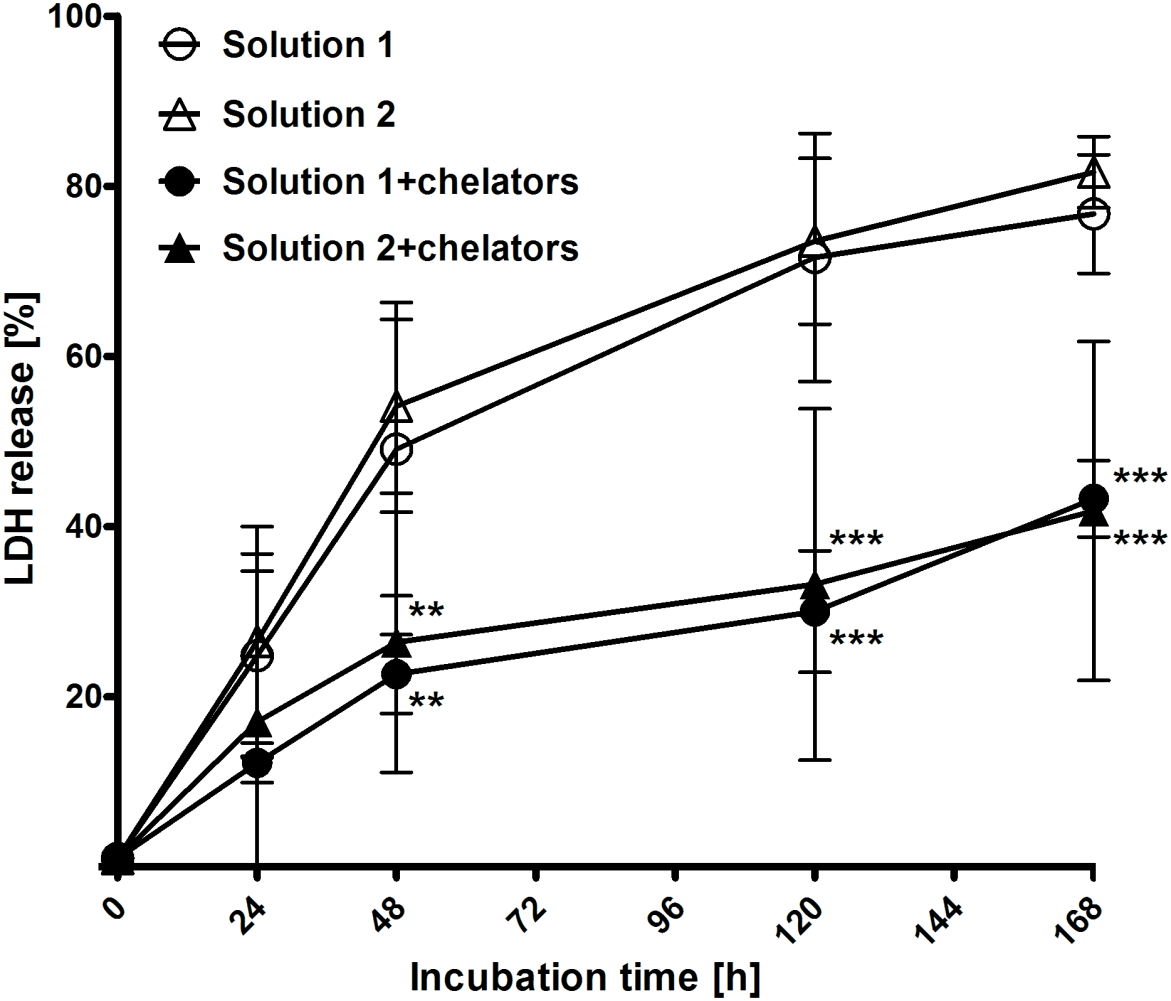
LDH release of isolated proximal renal tubules during cold incubation at 4°C for 168 h. Isolated tubules were incubated at 4°C in the chloride-rich solution 1 or its chloride-poor counterpart solution 2 in the absence or presence of the iron chelators deferoxamine (0.5 mM) and LK 614 (20 μM). Results display released LDH activity as a percentage of total LDH activity. Values are means ± standard deviation of tubules of five preparations. **p <0.01 and ***p <0.001 vs. respective solution without iron chelators.

Hypothermic injury. Hypothermic injury to isolated proximal renal tubules, as assessed by LDH release, a quantitative marker of cell death, was followed during cold incubation (4°C) for 168 h. In solution 1 LDH release of isolated proximal renal tubules during cold incubation at 4°C continuously increased during the course of the experiment reaching a maximum of 77 ± 7% after 168 h (Fig 1). No significant differences in LDH release could be found between solution 1 and the chloride-poor variant solution 2. Addition of iron chelators resulted in a significantly lower LDH release in both solutions after 48 h of cold incubation (p <0.01; n = 5) with further increase of differences over time (after 120 h and 168 h: p <0.001; n = 5). No significant differences in LDH release between iron chelator-containing solutions with high and low chloride concentrations could be found.

**Fig 2:**
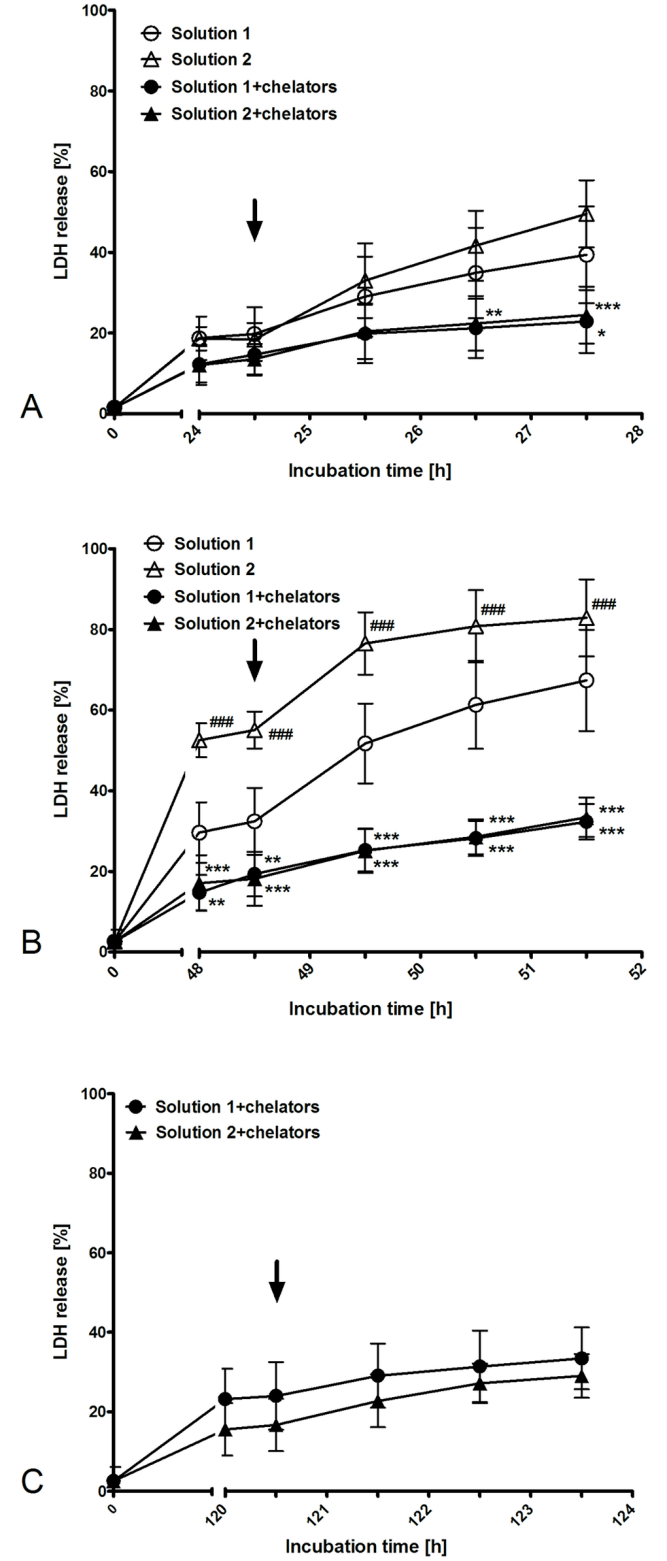
LDH release of isolated proximal renal tubules during rewarming after 24 h, 48 h and 120 h of cold incubation. Isolated tubules were incubated at 4°C in the chloride-rich solution 1 or its chloride-poor counterpart solution 2 in the absence or presence of the iron chelators deferoxamine (0.5 mM) and LK 614 (20 μM) for 24 h (A), 48 h (B) or 120 h (C). For gentle rewarming cell suspensions in the respective cold solutions were kept at room temperature for 10 min followed by 10 min at 37°C (transition period). Afterwards, tubules were spun down, resuspended in pre-warmed extra-cellular buffer and incubated under gentle motion at 37°C for three hours. Arrows mark time of buffer exchange. Results display released LDH activity as a percentage of total LDH activity. Values are means ± standard deviation of tubules of three (A), eight (B) and eight (C) preparations. (A) *p <0.05, **p <0.01 and ***p <0.001 vs. respective solution without iron chelators. (B) ###p <0.001 vs. solution 1. **p <0.01 and ***p <0.001 vs. respective solution without iron chelators.

Rewarming after 24 h of cold incubation. Rewarming of isolated proximal renal tubules in extra-cellular buffer after 24 h of cold incubation (4°C) in solution 1 and the successive transition period resulted in moderate aggravation of the injury (Fig 2A). No significant differences in LDH release could be found between solution 1 and the chloride-poor variant solution 2. The addition of iron chelators during cold incubation (solution 1+chelators, solution 2+chelators) attenuated injury during rewarming (p <0.05; n = 3). No significant differences in LDH release between iron chelator-containing solutions with high and low chloride concentrations could be found.

Rewarming after 48 h cold incubation. Rewarming of isolated proximal renal tubules in extra-cellular buffer after 48 h of cold incubation (4°C) in solution 1 plus the successive transition period resulted in marked aggravation of injury (Fig 2B). Similarly, rewarming caused aggravation of injury after cold incubation in the chloride-poor variant solution 2. In this set of experiments (n = 8, i.e. lower standard deviation) LDH release after 48 h of cold incubation in solution 2 was, (in contrast to the data shown in Fig 1) already significantly higher than after cold incubation in solution 1 (p <0.001; n = 8); this difference remained during rewarming. In both solutions, addition of iron chelators during cold incubation resulted in a lower LDH release after 48 h of cold incubation (p <0.01), and even more so during rewarming (p <0.001). No significant differences in LDH release between iron chelator-containing solutions with high and low chloride concentrations could be found.

Rewarming after 120 h of cold incubation. As after 120 h of cold incubation tubules cold-incubated in the absence of iron chelators were predominantly already dead by the end of cold incubation (Fig 1), the effect of rewarming was only assessed after cold incubation in solutions containing iron chelators. Rewarming after cold incubation in both chelator-containing solutions only marginally increased LDH release (Fig 2C). No significant differences between solution 1+chelators and solution 2+chelators could be found.

**Fig 3:**
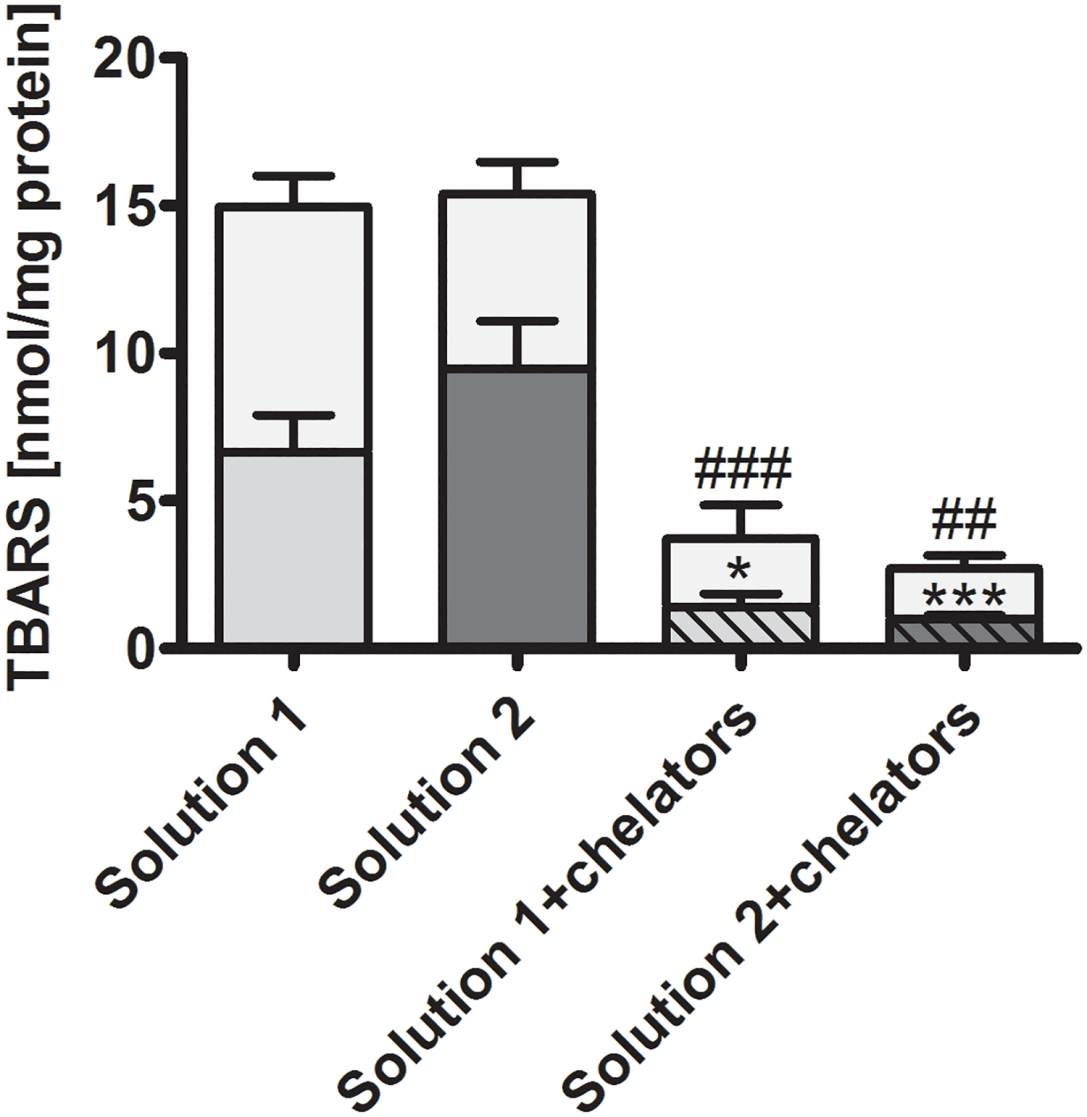
Lipid peroxidation in isolated proximal renal tubules after cold incubation and rewarming. Isolated tubules were incubated at 4°C in the chloride-rich solution 1 (intermediate grey bars) or its chloride-poor counterpart solution 2 (dark grey bars) each in the absence or presence of the iron chelators deferoxamine (0.5 mM) and LK 614 (20 μM; striped bars) for 48 h under gentle agitation. Thereafter, tubules were spun down, resuspended in pre-warmed extracellular buffer and incubated under gentle motion at 37°C for two hours. Thiobarbituric acid-reactive substances (TBARS) were determined in the supernatants at the end of cold incubation (darker part of the bars) and at the end of rewarming (light grey part of the bars). *p<0.05 and ***p<0.001 vs. respective solution without iron chelators at the end of cold incubation; ##p<0.01 and ###p<0.001 vs. respective solution without iron chelators at the end of rewarming (n = 7).

Lipid peroxidation. TBARS, a marker of lipid peroxidation, i.e. of oxidative membrane injury, amounted to about 7 nmol/mg protein after 48 h of cold incubation in solution 1 and approx. doubled during rewarming (Fig 3). Values after cold incubation in solution 2 were not significantly different from values after cold incubation in solution 1. The addition of the iron chelators to the cold storage solution significantly inhibited TBARS formation in both solutions during cold incubation as well as during rewarming.

**Fig 4:**
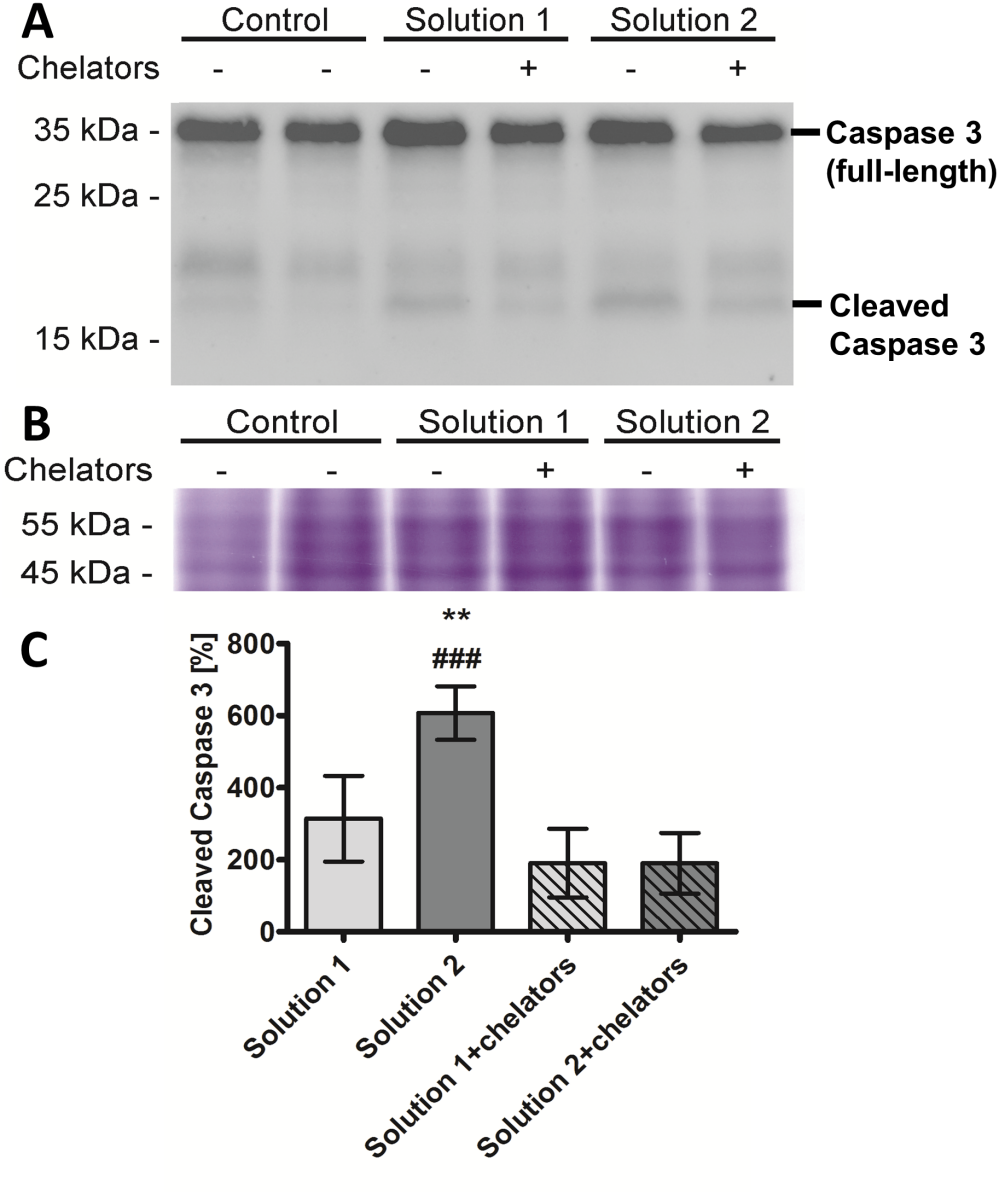
Caspase 3 cleavage of isolated renal proximal tubules following rewarming after 48 h of cold incubation. Isolated tubules were incubated at 4°C in chloride-rich solution 1 or its chloride-poor counterpart solution 2 each in the absence and presence of the iron chelators deferoxamine (0.5 mM) and LK 614 (20 μM) for 48 h. Tubule suspensions were rewarmed in extra-cellular buffer at 37°C for two hours. Total protein extracts of renal proximal tubules were prepared using RIPA buffer and equal amounts of protein (40 μg) were separated by SDS-PAGE and transferred onto nitrocellulose membrane. Full-length and cleaved caspase 3 was assessed in tubules of four preparations using a rabbit antibody against caspase 3. (A) Representative Western Blot for caspase 3 and cleaved caspase 3. (B) Loading control. Equal amounts of total protein (8 μg) of the protein extracts were separated by SDS-PAGE and coomassie staining was performed to check for comparable loading. (C) Quantification of cleaved caspase 3 (expressed as percentage of the respective signal of non-stored control tubules; n = 4). ** p<0.01 vs. solution 1. ### p<0.001 vs. solution 2+iron chelators.

Caspase 3 cleavage. Caspase 3 cleavage as a marker for apoptosis was generally relatively low (Fig 4A). A low degree of caspase 3 cleavage was already seen in the freshly isolated control tubules. Cold incubation in solution 1 and even more so in the chloride-poor solution 2 for 48 h followed by rewarming (2 h) increased caspase 3 cleavage (Fig 4A and 4C). The increase in caspase 3 cleavage after cold incubation in solution 1 (as compared to non-stored control tubules) was not statistically significant, but the increase observed after cold incubation in solution 2 was significant both compared to non-stored control tubules and to tubules cold-incubated in solution 1 (Fig 4C). In both solutions, addition of iron chelators during cold storage almost completely inhibited caspase 3 cleavage observed after rewarming, an effect that was statistically significant in solution 2.

**Fig 5:**
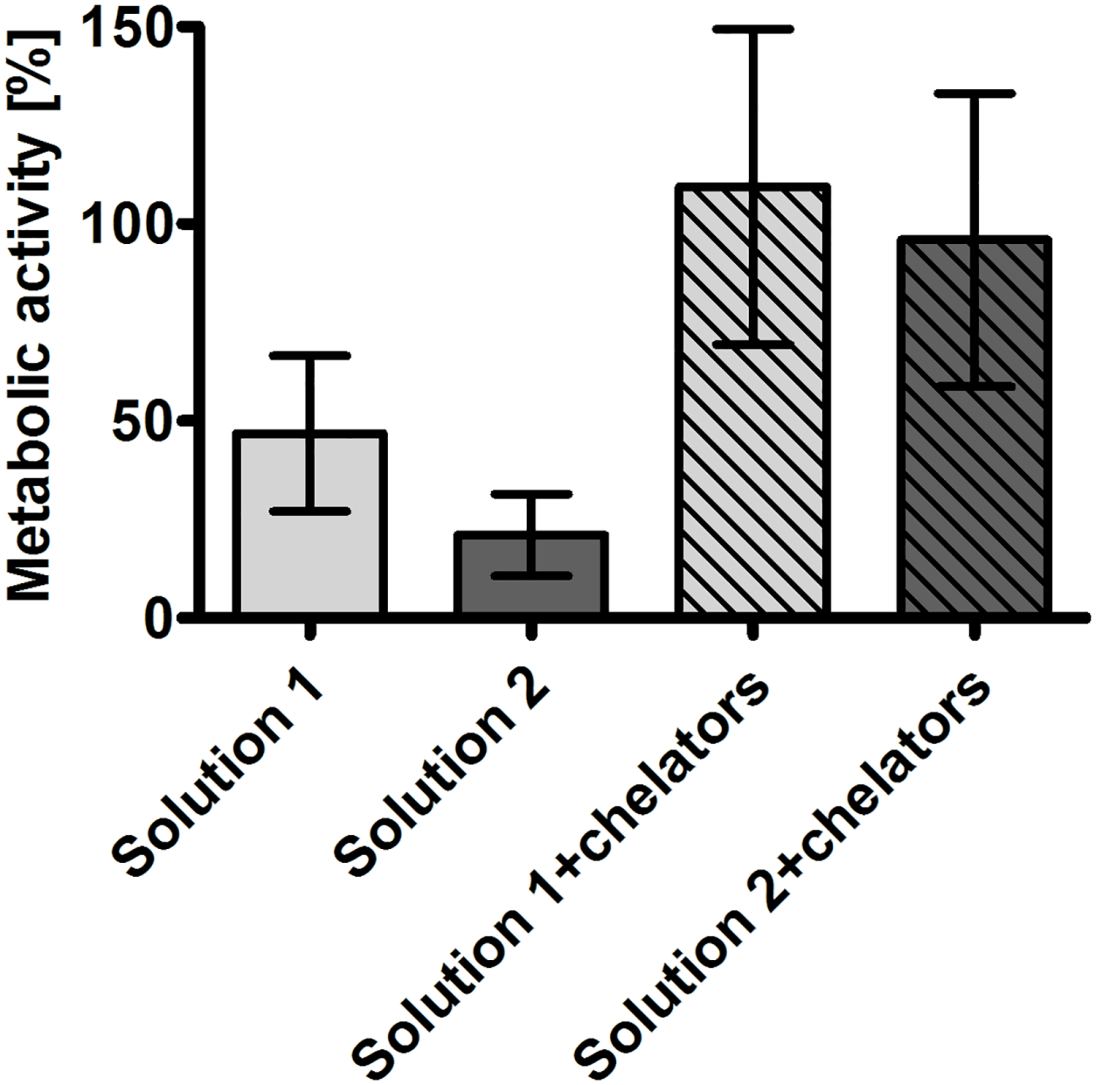
Metabolic activity of isolated renal proximal tubules following rewarming after 48 h of cold incubation. Isolated tubules were incubated at 4°C in the chloride-rich solution 1 or its chloride-poor counterpart solution 2 in the absence or presence of the iron chelators deferoxamine (0.5 mM) and LK 614 (20 μM) for 48 h. Tubule suspensions were rewarmed in extra-cellular buffer at 37°C for three hours; thereafter, metabolic activity was assessed by the resazurin reduction assay. Reduction of resazurin to fluorescent resorufin was followed at λ_exc_ = 560 nm, λ_em_ = 590 nm. Reduction rates are given as percentage of non-stored control tubules. Values are means ± standard deviation of tubules of four preparations.

Metabolic activity. Metabolic activity as indicated by resazurin reduction was decreased to about 50% of that of non-stored control tubules after 48 h of cold incubation in solution 1 plus rewarming (Fig 5). Metabolic activity tended to be even lower after cold incubation in solution 2. Addition of iron chelators during cold incubation maintained metabolic activity at the level of non-stored control tubules (109 ± 35% and 96 ± 37%; n = 4), although the difference to the incubations without iron chelators did not reach statistical significance. No significant differences in metabolic activity between iron chelator-containing solutions with high and low chloride concentrations could be found.

**Fig 6:**
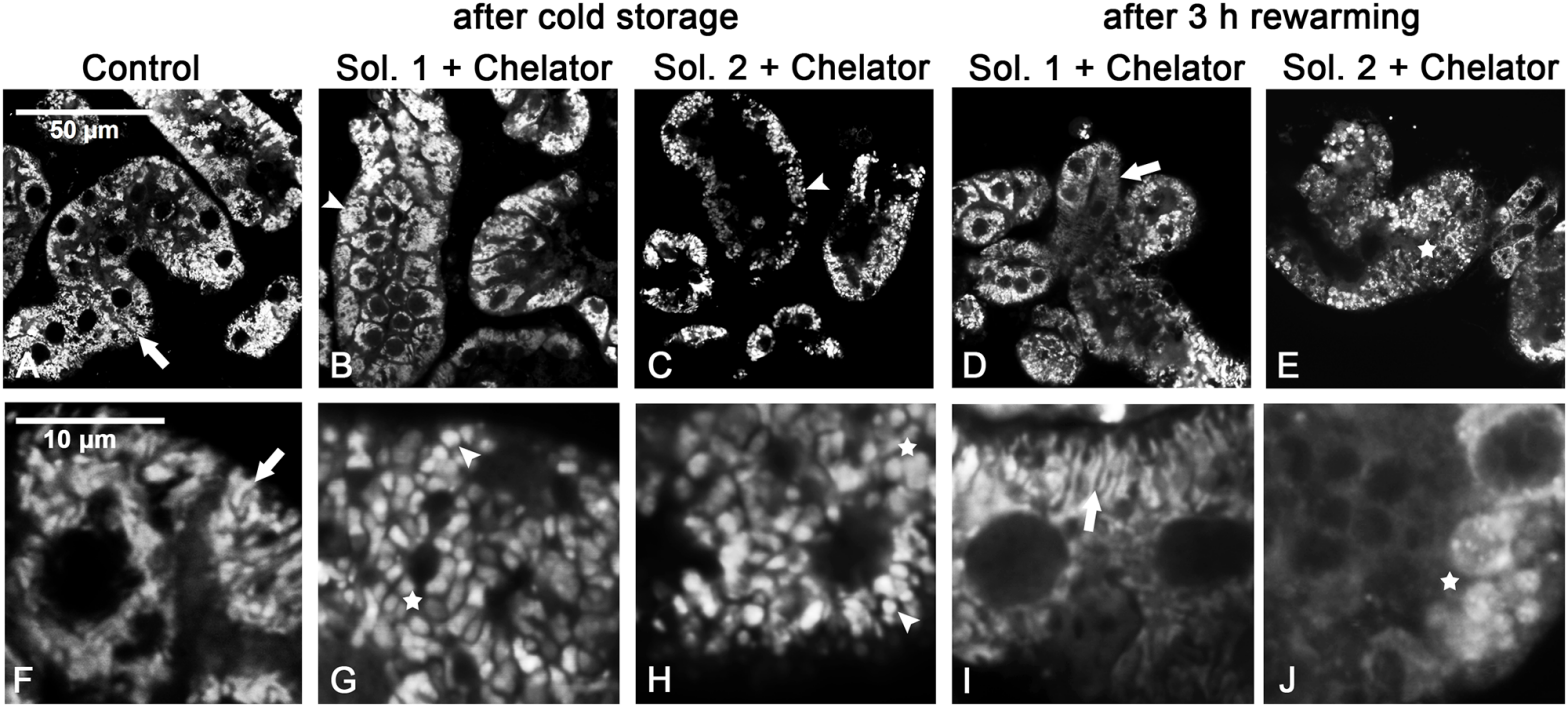
Mitochondrial alterations after cold incuabtion and rewarming. Freshly isolated rat proximal renal tubules (A, F: control) were incubated at 4°C in chloride-rich (B, D, G, I) or chloride-poor (C, E, H, J) cold storage solution with addition of iron chelators for 48 h. Mitochondria were stained with 300 nM MitoTracker Red at the beginning of cold storage and confocal laser scanning images were taken after fixation at the end of cold storage (B, C; detail: G, H) or after 3 h of rewarming (D, E; detail: I, J). Arrows: filamentous mitochondria; arrow heads: mitochondrial fragmentation; stars: mitochondrial swelling.

Tubular and mitochondrial morphology. During rewarming after cold incubation in all solutions, tubules showed bleb formation. Control tubules were rich in mitochondria, which predominantly showed a filamentous morphology and were located in the basal part of the tubules (Fig 6A; detailed view in Fig 6F; arrows). After cold incubation for 48 h, mitochondrial fragmentation (Fig 6B, 6C, 6G and 6H, see arrowheads) and some degree of mitochondrial swelling (Fig 6G and 6H; stars) could be observed in all solutions. During rewarming, mitochondria of part of the tubules, especially after cold incubation in solution 1+chelators partially regained their filamentous morphology (Fig 6D; detailed view in Fig 6I, arrows), while mitochondria in other tubules (not only under the most injurious conditions in the absence of iron chelators, not shown, but also in solution 2+chelators) predominantly showed persistent fragmentations and/or gross swelling (Fig 6E; detailed view in Fig 6J, stars). However, we have to note that, although behavior was relatively homogenous within the individual tubule, the freshly isolated tubules were heterogeneous and a mixture of different morphologies (with only different predominances) was seen under all incubation conditions.

**Fig 7:**
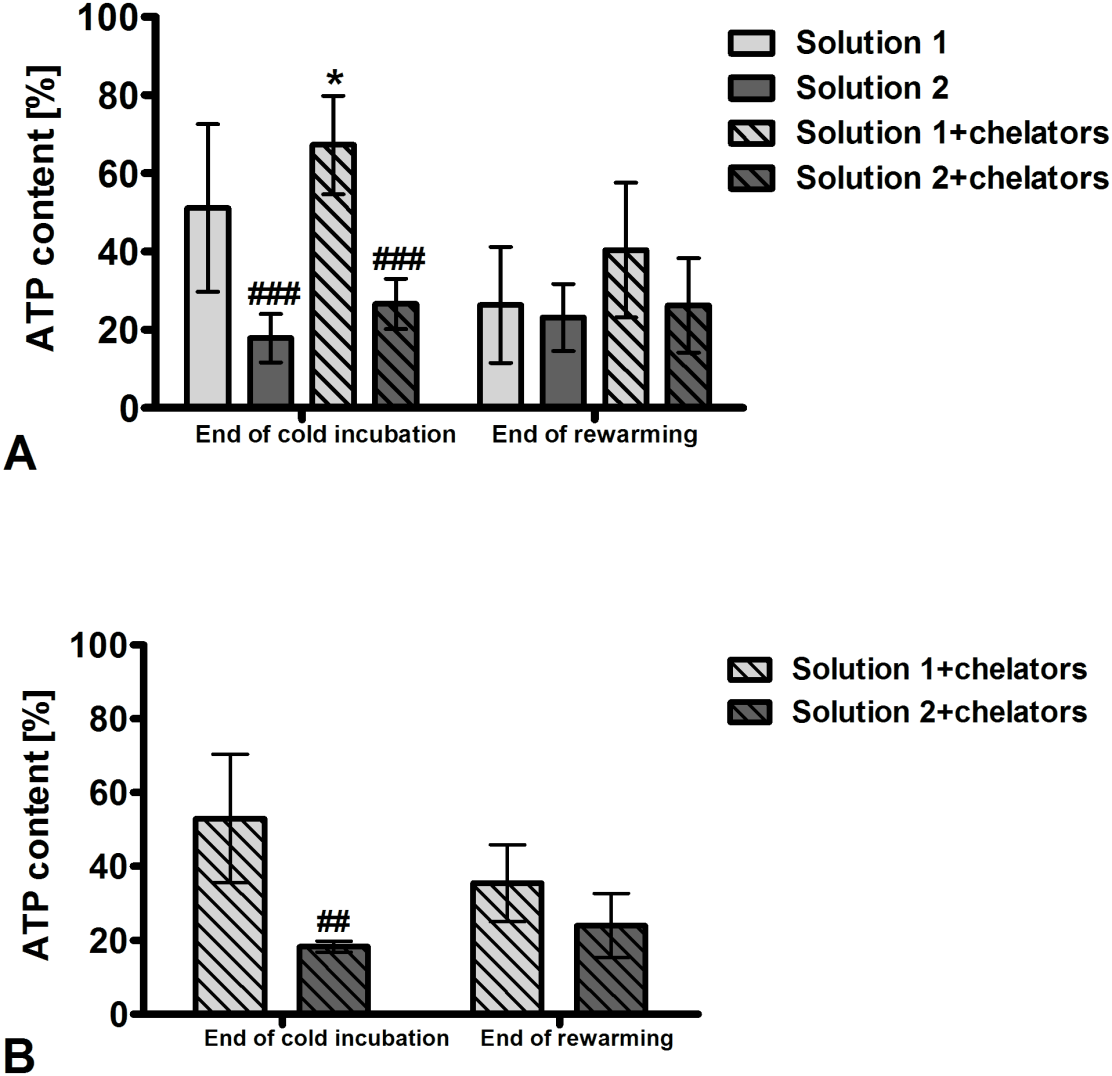
ATP content of isolated renal proximal tubules after cold incubation and subsequent rewarming. Isolated tubules were incubated at 4°C in the chloride-rich solution 1 or its chloride-poor counterpart solution 2 in the absence or presence of the iron chelators deferoxamine (0.5 mM) and LK 614 (20 μM) for 48 h (A) and 120 h (B). Tubules were rewarmed in extra-cellular buffer at 37°C for three hours. ATP content was normalized for protein content and is expressed as percentage of the respective non-stored control tubules. (A) *p <0.05 vs. solution 1; ###p <0.001 vs. respective chloride-rich solution. (B) ##p <0.01 vs. solution 1+chelators. Values are means ± standard deviation of eight (A) and four (B) preparations.

Energy state after cold incubation and rewarming. ATP content of renal proximal tubules measured directly after isolation was 5.5 ± 1.6 nmol/mg protein. Cold incubation for 48 h decreased ATP content in all solutions. After cold incubation in solution 1 ATP content was decreased to about 50% of baseline values (Fig 7A). It was even further decreased after 48 h of cold incubation in the chloride-poor solution 2 (p <0.001; n = 8). Addition of iron chelators to the cold incubation solutions led to a slightly higher ATP content at the end of cold incubation, which was significant for solution 1+chelators (p <0.05; n = 8). During rewarming, there was a further decline of ATP content in the so far best conditions (solution 1 and solution 1+chelators) and only marginal alterations under the other conditions so that ATP content was below 40% of that of control tubules under all conditions.

After 120 h of cold incubation in solution 1+chelators, ATP content was also significantly better preserved than after cold incubation in solution 2+chelators (p <0.01; n = 4) (Fig 7B). During rewarming ATP content of tubules incubated in solution 1+chelators again decreased, while ATP content in solution 2+chelators marginally increased.

**Fig 8:**
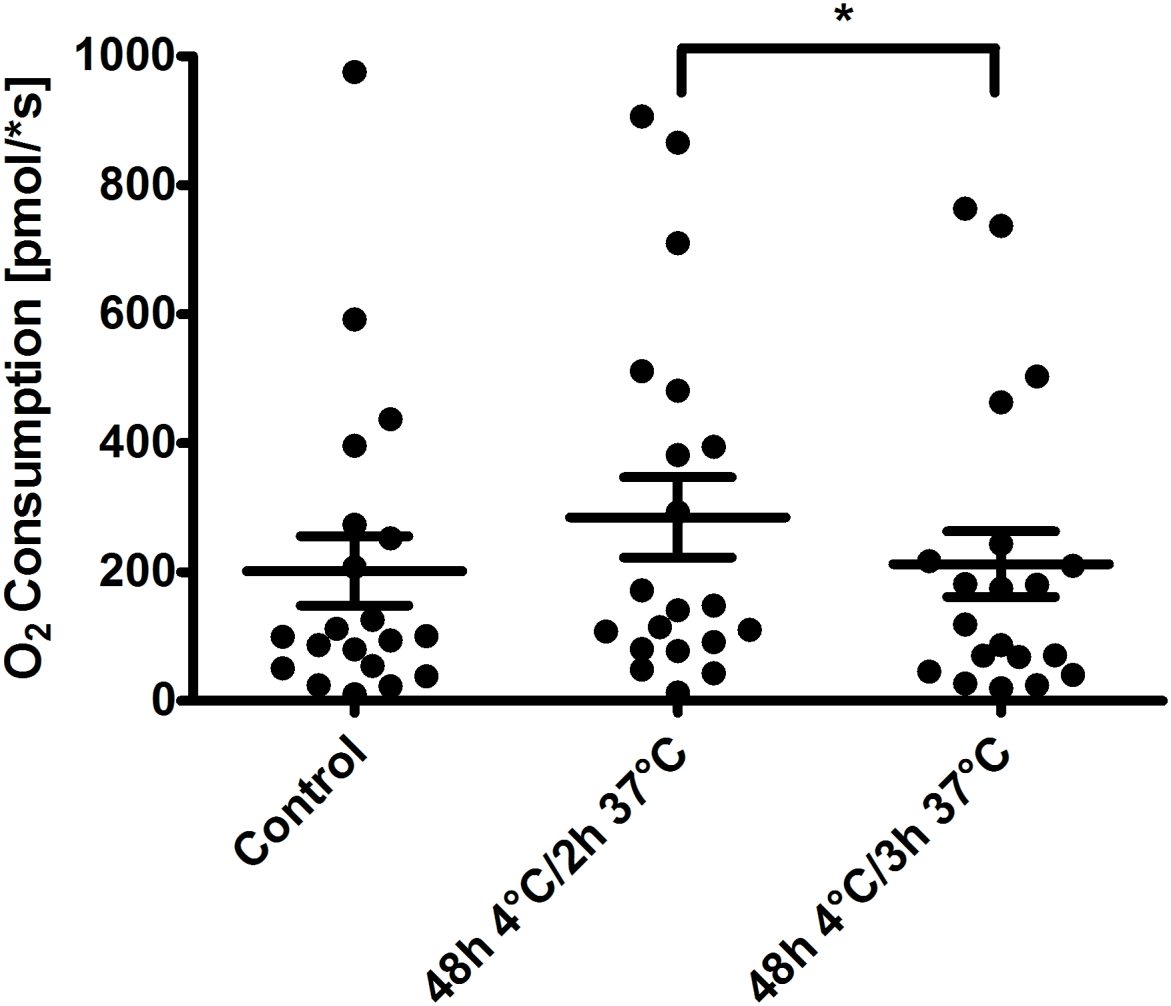
Oxygen consumption of renal proximal tubules. Isolated tubules were incubated at 4°C in the chloride-rich solution 1 in the presence of the iron chelators deferoxamine (0.5 mM) and LK 614 (20 μM) for 48 h. Tubules were rewarmed in extra-cellular buffer at 37°C for up to three hours. Oxygen consumption was measured at baseline (control) and after 48 h of cold incubation followed by 2 h or 3 h of rewarming in extra-cellular buffer. Respiration was measured in extra-cellular buffer at 37°C under continuous stirring using an O2K-oxygraph. Results summarize data from 20 measurements. Two measurements each were performed with utilization of tubules from 10 individual preparations. *p <0.05.

Oxygen consumption after 48 h of cold incubation and rewarming. Oxygen consumption of primary renal proximal tubules was highly variable. Oxygen consumption in freshly isolated renal proximal tubules measured at 37°C was 202 ± 240 pmol/s for an aliquot of tubules corresponding to about 2 mg of protein. After cold incubation at 4°C for 48 h in solution 1+chelators and subsequent rewarming for 2 h oxygen consumption did not decrease, but showed a tendency to increase. After 3 h of rewarming oxygen consumption was significantly lower than after 2 h of rewarming (p <0.05; n = 20) (Fig 8). No differences of oxygen consumption between tubules incubated in solution 1+chelators or 2+chelators could be detected after 2 h or 3 h of rewarming (n = 10; data not shown).

Detailed results of all experiments performed are shown as supporting information (S1 File).

## Discussion

Results of the present study confirmed an iron-dependent component of preservation injury during cold incubation and rewarming in rat proximal renal tubules and revealed a benefit of chloride for the maintenance of tubular energy state during cold incubation.

Experimental results confirmed a marked improvement of cellular integrity (as indicated by reduced LDH release) by addition of iron chelators to cold incubation solutions. This effect became obvious in the present study after 48 h of cold incubation (Fig 1) and persisted during rewarming (Fig 2B). Lipid peroxidation, i.e. oxidative membrane injury, during both, cold incubation itself and during rewarming was also decreased by the addition of iron chelators (Fig 3). Metabolic activity in tubules incubated in the presence of iron chelators was also tendentially better preserved (Fig 5) and similar to non-stored control tubules. The beneficial effects of iron chelators on cold-induced cellular damage is well established for diverse cell types [7, 22, 32, 33] and also for renal tubular cells and tubules [8, 19, 20]. Also caspase 3 activation during rewarming after cold storage (Fig 4) has been described and has been found to be inhibited by iron chelators [19]. Hypothermia has been shown previously to induce a rapid increase in the cellular chelatable iron pool that causes lipid peroxidation and mitochondrial permeability transition both resulting in enhanced cell death [7, 18, 19, 32, 33].

However, addition of iron chelators to the cold storage solutions had only moderate impact on energy state indicated by ATP content (Fig 7). In contrast, the inorganic anion chloride showed a distinct influence on maintenance of cellular energy state: chloride-rich solutions were highly beneficial for energy preservation during cold incubation. The effects of chloride and of solutions low in chloride on cold-induced damage have been studied in rat hepatocytes [16, 22], porcine aortic segments [23], a mouse cardiac [34], and a rat liver transplant model [35], whereas data on renal tubules or the kidney were completely missing. As a limitation, freshly isolated rat renal proximal tubules showed moderate inter-preparation differences with regard to tubule number, post-preparational energy content and O_2_ consumption, but behavior regarding tubular energy state was reproducible. Our finding of better preserved energy content in chloride-rich solutions after cold incubation contradicts with the finding of better maintenance of viability in chloride-poor solutions in rat hepatocytes [16, 22]. Although sodium concentration and cellular swelling do not impact cold-induced injury of rat hepatocytes [9], chloride does provoke impairment [16, 22]. Chloride-induced impairment of rat hepatocytes protected by an iron chelator has been described to occur predominantly during rewarming [22]. On the other hand, there are cells in which, in line with the energy state observed here, the iron-*in*dependent component of damage is intensified in the absence of chloride. Very similar to rat renal tubules, aortic endothelial cells [23] and human hepatocytes [27] prefer chloride-rich preservation solutions during cold incubation. Cold incubation in the absence of chloride decreased tubular ATP content in the current study (Fig 7). This mitochondrial impairment elicited by cold storage in chloride-poor solutions is, notably, independent on the previously described iron-dependent mitochondrial injury during cold storage (iron-dependent mitochondrial permeability transition, see above) as this injury also occurred in the presence of iron chelators (Fig 7; in concentrations sufficient to inhibit cold-induced cell death, compare Fig 1). By which mechanism chloride-poor solutions trigger mitochondrial injury is currently unknown. Although mitochondria display chloride channels in the inner mitochondrial membrane, it is largely unclear which role chloride plays in mitochondria [36].

The tendency of increased oxygen consumption following cold incubation and 2 h of rewarming in comparison to non-stored control tubules (Fig 8) suggests, especially with regard to the reduced amount of vital tubules participating in cellular metabolism and the reduced ATP content, a certain degree of uncoupling of the respiratory chain (or, alternatively, increased need for ATP e.g. within the context of repair processes). As no differences in oxygen consumption between chloride-rich and chloride-poor solutions could be observed, mechanisms leading to the lower energy content after cold incubation in the chloride-poor solutions remain unclear. The declining oxygen consumption after 3 h of rewarming might be expression of increasing mitochondrial or cellular injury. Measurement of oxygen consumption contains a random error as exact tubular concentration is difficult to adjust and as mechanical stress during measurement might also have an influence. This error was partly corrected for by the high number of performed measurements.

Cold, similar to hypoxia, has been shown to lead to a partial loss of mitochondrial membrane potential [24]. Additionally, a cold-induced fragmentation of mitochondria as present in renal proximal tubules was also observed in rat hepatic endothelial cells [24], human hepatocytes [27] and porcine aortic endothelial cells. In the presence of iron chelators and with moderate cold incubation time cold-induced mitochondrial fission was found to be reversible [24, 27]. In the present study mitochondrial fragmentation was partially reversible with inferior improvement in chloride-free solutions showing aggravated mitochondrial swelling. Merging (fusion) and division (fission) of mitochondria are essential remodeling processes involved in the adaptation of mitochondria to the cellular metabolic state [37, 38]. On the one hand, fission is not necessarily a feature of mitochondrial or cellular damage, but also essential during mitosis and involved in mitochondrial quality control [39]. On the other hand, fusion is known to enable enhanced energy production [37, 39]. With increased duration of cold incubation or inadequate protection mitochondria stayed fragmented with limited ability for energy provision. Mitochondrial changes including fragmentation and swelling were also obvious in renal tubules following cold incubation and partially reversible during rewarming. The tendency for poorer mitochondrial recovery in chloride-poor solutions appears as a potential starting point for further studies on mechanisms of mitochondrial injury induced by chloride-poor solutions. However, as primary proximal tubules are heterogeneous, isolated tubular cells or tubular cell lines might be a more suitable experimental model to study these mechanisms in detail.

## Conclusions

The present study suggests that cellular integrity (membrane integrity) is only one point that has to be addressed in the attempt to decrease storage-associated damage. Once early cell death is prevented, cellular and especially mitochondrial functionality gain importance. While LDH release was only 32% after rewarming in tubules incubated for 48 h in the chloride-rich solution containing iron chelators, reflecting about 68% of intact cells, ATP content was decreased to as little as 40% of baseline values. Iron-*in*dependent components of damage appear to impact especially mitochondria and thereby energy state. This should be considered with regard to reconditioning of organs after cold storage in which the re-establishment of normal energy metabolism is a major focus. The contrary behavior of tubules cold-incubated in chloride-poor solutions to show a slight tendency to increase energy content during rewarming, while energy loss proceeded to decline in tubules incubated in chloride-rich solutions, in this context requires further studies.

The iron-dependent component of hypothermic injury can be inhibited by iron chelators as implemented in the new preservation solution Custodiol-N [40, 41] and the tissue preservation solution TiProtec^®^ [23, 42]. The second component of injury induced by chloride-deficient solutions contradicts with older views on preservation-induced injury (see introduction), appears to be cell-type specific, seems to interfere with the cellular energy metabolism, and shows further unexplained features during rewarming. These results might challenge the use of chloride-poor preservation solutions as the standard for all tissues, and warrant further investigations into the underlying mechanisms to ensure a good energy state enabling optimal graft repair and function.

## Supporting information

S1 File Raw data. Detailed results of all experiments performed.
(XLSX)
